# How to identify IgA nephropathy presenting as nephrotic syndrome coexisting with minimal change disease? A 15-year single-center clinicopathological analysis

**DOI:** 10.3389/fimmu.2025.1669276

**Published:** 2025-11-06

**Authors:** Yue Yang, Lu-xian Duan, Ying Wang, Zheng Zhang, Qian-qian Xu, Li Zhuo, Wen-ge Li

**Affiliations:** 1 Department of Nephrology, China-Japan Friendship Hospital, Beijing, China; 2 Peking University China-Japan Friendship School of Clinical Medicine, Beijing, China

**Keywords:** IgA nephropathy, nephrotic syndrome, minimal change disease, anti-nephrin autoantibodies, corticosteroid responsiveness

## Abstract

**Objectives:**

Nephrotic syndrome (NS) in IgA nephropathy (IgAN) may indicate concurrent minimal change disease (MCD). This study characterized the IgAN-MCD overlap phenotype using anti-nephrin autoantibodies (IgG co-localization) in NS-IgAN patients, assessing its prevalence and therapeutic implications.

**Methods:**

We conducted a retrospective analysis of 67 biopsy-confirmed NS-IgAN patients (2010-2024) with ≥1-year follow-up. Patients were stratified by treatment response into complete remission (CR, n = 24) and non-remission (NR, n = 26) groups. Renal biopsies were evaluated for anti-nephrin autoantibodies via IgG co-localization and podocyte ultrastructure. Longitudinal data were analyzed using repeated-measures ANOVA with Benjamini-Hochberg correction; time-to-remission was assessed by Kaplan-Meier and Cox regression analyses.

**Results:**

CR patients showed significantly lower baseline serum albumin (18.8 ± 4.0 vs. 24.1 ± 4.2 g/L, *P* < 0.001) and higher eGFR (101 ± 29 vs. 62 ± 35 mL/min/1.73m², *P* < 0.001) compared to NR patients. Anti-nephrin IgG co-localization was detected in 54.2% of CR patients but absent in NR patients (*P* < 0.001). Cox regression identified anti-nephrin positivity as a strong predictor of faster remission (HR: 0.40, 95% CI: 0.17-0.90; *P* = 0.028). CR patients achieved rapid proteinuria remission (0.1 ± 0.1 vs. 7.0 ± 0.8 g/24h at 1 month, *P* < 0.001) with significant time×group interactions for proteinuria (*P* = 0.001) and serum albumin (*P* = 0.004). An estimated 35.8% of NS-IgAN cases represented IgAN-MCD overlap.

**Conclusion:**

A significant subset (~36%) of NS-IgAN patients exhibit an IgAN-MCD overlap state identifiable by renal anti-nephrin IgG co-localization, demonstrating MCD-like pathology and excellent corticosteroid response. This biomarker integration can guide personalized therapy, enabling effective short-course treatment for overlap cases while avoiding unnecessary long-term immunosuppression in classic NS-IgAN.

## 1 Introduction

IgA nephropathy (IgAN) is the most common primary glomerulonephritis worldwide, with particularly high prevalence in Asian populations (1). The diagnosis of IgAN relies on renal biopsy. In China, IgAN accounts for approximately 45.3% of all primary glomerular diseases (2). Notably, 30-40% of adult IgAN patients progress to end-stage renal disease (ESRD) within 20–30 years after diagnosis (1, 3, 4). The clinical manifestations exhibit substantial heterogeneity, including episodic macroscopic hematuria, asymptomatic microscopic hematuria with or without proteinuria, and renal dysfunction. Nephrotic syndrome (NS), characterized by heavy proteinuria (>3.5 g/24h) and hypoalbuminemia (serum albumin <30 g/L), is considered an uncommon clinical presentation of IgAN (5), observed in only 6.7-14.7% of patients at disease onset (6–9).

As early as 2014, Herlitz et al. (10) documented rare cases of mild IgAN with nephrotic-range proteinuria that exhibited clinicopathological features, therapeutic responses, and outcomes suggestive of IgAN superimposed with minimal change disease (MCD), proposing these as dual glomerulopathies. However, the absence of validated MCD biomarkers at that time limited conclusive evidence beyond clinical correlations. The 2021 KDIGO guidelines acknowledge that IgA nephropathy with nephrotic syndrome (NS-IgAN) shares podocytopathic features with MCD, and thus recommend that patients demonstrating mesangial IgA deposition with MCD-compatible ultrastructural features should receive therapy per MCD treatment protocols (5). Nevertheless, critical unresolved challenges persist: 1) how to reliably differentiate IgAN-MCD overlap from NS-IgAN; 2) the epidemiological prevalence of this dual glomerulopathy among NS-IgAN patients; and 3) what definitive histopathological or serological criteria confirm concurrent IgAN-MCD. These questions retain clinical significance due to substantial disparities in therapeutic responses and long-term renal outcomes.

Recent investigations have identified anti-nephrin autoantibodies as pivotal mediators in the pathogenesis of podocytopathies, particularly MCD (11–13). In a landmark multicenter study, Hengel et al. (11) established a novel quantitative method for detecting circulating anti-nephrin antibodies, demonstrating a strong correlation between antibody titers and MCD disease activity. Furthermore, Watts and colleagues (12) elucidated the mechanistic basis of podocyte injury in MCD through immunofluorescence co-localization studies, revealing spatial congruence between punctate IgG deposits and nephrin protein distribution within glomeruli.

This retrospective cohort study analyzed clinical characteristics, pathological profiles, and prognostic heterogeneity in NS-IgAN patients diagnosed at our center between 2010 and 2024. Renal biopsy specimens were re-evaluated using novel molecular biomarkers through immunofluorescence analysis. This study aims to further enhance understanding of the IgAN-MCD overlap state, clarify the utility of these biomarkers in identifying IgAN-MCD patients among NS-IgAN cases, provide evidence for personalized treatment strategies, avoid unnecessary long-term use of corticosteroids/immunosuppressants, minimize drug-related adverse effects while ensuring therapeutic efficacy, and maximize clinical benefits for patients.

## 2 Methods

### 2.1 Study design and inclusion/exclusion criteria

This retrospective cohort study screened 1,298 patients with biopsy-proven primary IgAN at China-Japan Friendship Hospital between January 2010 and December 2024. Inclusion criteria comprised: 1) meeting NS diagnostic criteria (24-hour urinary total protein [24hUTP] >3.5 g and serum albumin <30 g/L); 2) age 18–75 years; 3) minimum follow-up duration of 12 months. Exclusion criteria included: 1) renal biopsy specimens containing fewer than 8 glomeruli; 2) baseline estimated glomerular filtration rate (eGFR) ≤15 mL/min/1.73m²; 3) secondary IgAN (e.g., Henoch-Schönlein purpura); 4) comorbid systemic diseases affecting renal function (e.g., diabetes mellitus, viral hepatitis, malignancies). After rigorous screening, 67 patients qualified for analysis.

Based on proteinuria response following standardized initial therapy, patients were stratified into: 1) complete remission (CR) group (achieving 24hUTP <0.3 g/24h at 1 month), 2) non-remission (NR) group (showing persistent 24hUTP >3.5 g/24h despite 3 months of therapy), and 3) partial remission (PR) group (comprising all other patients not meeting CR or NR criteria). Comprehensive retrospective analyses were conducted on clinical, pathological, and immunological parameters.

### 2.2 Data collection

Clinical data were systematically collected at the time of renal biopsy (baseline) and throughout the follow-up period. The data encompassed demographic characteristics (age, sex), blood pressure, body mass index (BMI), urinary sediment red blood cell count, 24-hour urinary total protein (24hUTP), serum albumin, serum creatinine, estimated glomerular filtration rate (eGFR, calculated using the CKD-EPI equation), serum lipids (total cholesterol, triglycerides), immunoglobulins, complement levels, and detailed pathological characteristics. Renal histopathological reports, including Oxford classification scores, were independently evaluated by two renal pathologists. Immunofluorescence deposition intensity was graded on a 5-tier scale (0 to 4+), while electron microscopy findings categorized foot process effacement as mild (<30% involvement), moderate (30-70%), or diffuse (>70%).

Treatment regimens were retrospectively analyzed, including corticosteroid, immunosuppressants, and renin-angiotensin-aldosterone system inhibitors (RAASi). Therapeutic responsiveness was assessed through serial 24hUTP measurements and eGFR monitoring.

### 2.3 Pathological examination

In addition to routine immunofluorescence and light microscopy examinations, renal biopsy specimens from all enrolled patients underwent ultrastructural evaluation via transmission electron microscopy (TEM) and targeted immunofluorescence analysis for specific biomarkers.

For TEM analysis, tissues were fixed in 2.5% glutaraldehyde, dehydrated, embedded, and sectioned into 70–90 nm ultrathin slices. Following double-staining with uranyl acetate and lead citrate, ultrastructural characteristics were examined using a Hitachi HT7800 TEM operated at 80 kV. Morphometric evaluation included systematic quantification of glomerular basement membrane (GBM) thickness and assessment of podocyte ultrastructure, with particular focus on foot process effacement and cytoskeletal integrity.

Immunofluorescence analysis was performed to detect podocyte-specific biomarkers. After antigen retrieval, tissue sections were incubated with primary antibodies against nephrin (R&D Systems), synaptopodin (Progen), podocin (Millipore Sigma), WT1 (Invitrogen), and IgG (Abcam). Fluorescent imaging was conducted using a Zeiss inverted fluorescence microscope under standardized optical configurations.

### 2.4 Statistical analysis

Data were analyzed using SPSS software (version 23.0; IBM Corp.) and GraphPad Prism (version 9.5; GraphPad Software), with all statistical tests being two-sided and a *P*-value <0.05 considered statistically significant. Continuous variables with normal distribution are expressed as mean ± standard deviation (SD) and analyzed using Student’s t-test for intergroup comparisons, while non-normally distributed continuous variables were presented as median (interquartile range, IQR) and compared via Mann-Whitney U test. Categorical variables were reported as numbers (percentages) and analyzed using Pearson’s chi-square test or Fisher’s exact test, as appropriate. Longitudinal data were analyzed using repeated-measures ANOVA to examine the main effects of time and group, as well as the time-by-group interaction effect, with results presented as mean ± standard error of the mean (SEM). Additionally, *post-hoc* power analysis was performed using G*Power software (version 3.1) to evaluate the statistical power of the study.

## 3 Results

### 3.1 Enrollment and patient stratification

**Figure 1:**
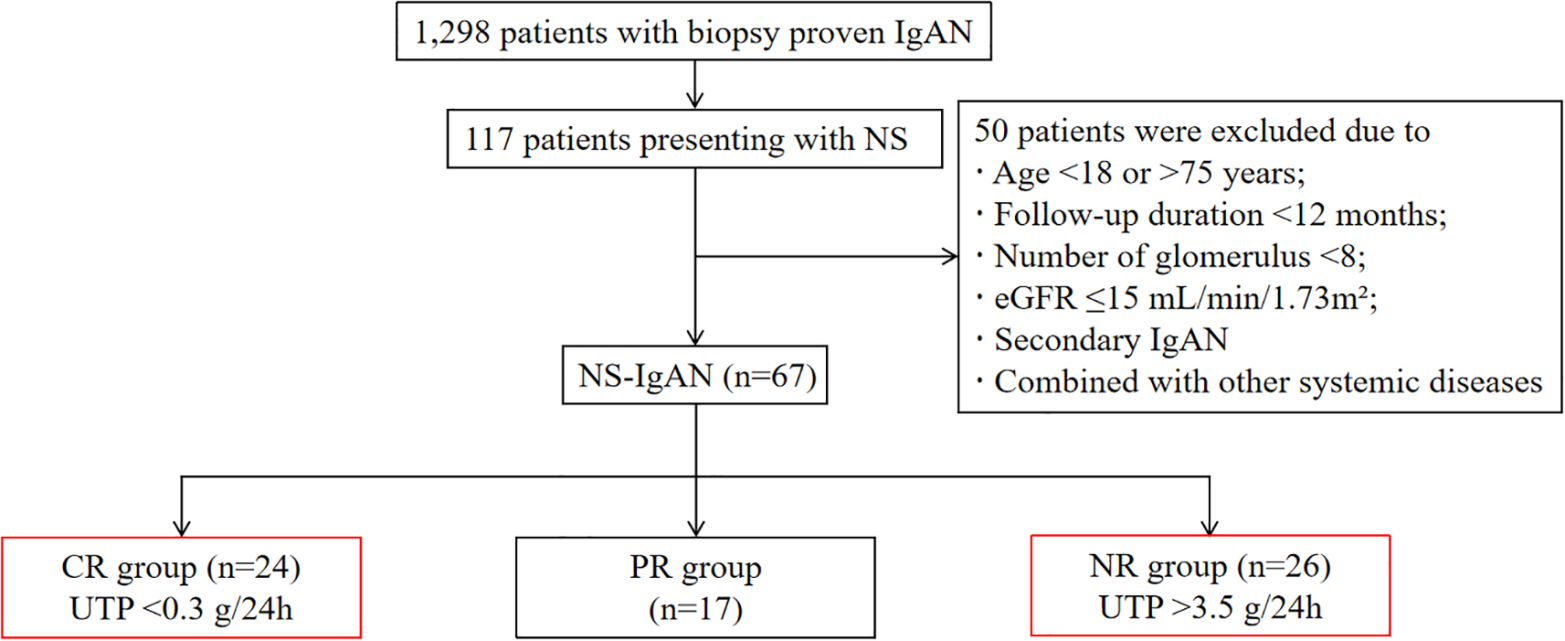
Flow chart of patient selection. IgAN, IgA nephropathy; NS, nephrotic syndrome; eGFR, estimated glomerular filtration rate; CR, complete remission; PR, partial remission; NR, non-remission; UTP, urinary total protein.

Among 1,298 biopsy-confirmed primary IgAN patients, 117 (9.0%) met the diagnostic criteria for NS (24hUTP >3.5 g/24h with serum albumin <30 g/L). Following therapeutic intervention, patients were stratified into three groups based on therapeutic response: the CR group comprised individuals achieving urinary protein remission (24hUTP <0.3 g/24h) within 1 month of treatment; the NR group included patients maintaining nephrotic-range proteinuria (24hUTP >3.5 g/24h) after 3 months of therapy; all remaining patients were classified into the PR group (**Figure 1**).

### 3.2 Clinical and pathological characteristics

As detailed in **Table 1**, the CR group exhibited significantly lower baseline serum albumin levels compared to the NR group (18.8 ± 4.0 vs. 24.1 ± 4.2 g/L, *P* < 0.001), whereas 24hUTP levels showed no intergroup difference. Notably, the CR group demonstrated superior renal function, with significantly higher baseline eGFR (101 ± 29 vs. 62 ± 35 mL/min/1.73m², *P* < 0.001). No significant disparities were observed in age, sex distribution, or BMI between the two cohorts. Pathological analysis based on the Oxford Classification revealed that the NR group exhibited more severe endocapillary hypercellularity (*P* < 0.001), higher prevalence of segmental glomerulosclerosis (*P* = 0.002), more advanced tubular atrophy/interstitial fibrosis (*P* = 0.005), and significantly increased crescent formation (*P* < 0.001) compared to the CR group. Immunofluorescence demonstrated significantly lower IgA (*P* = 0.015) and C3 (*P* < 0.001) deposition intensity in the CR group, while serum IgA and C3 levels showed no significant intergroup differences, with detailed baseline characteristics and pathological data for the PR group provided in the **Supplementary Materials**.

**Table 1:** Comparison of clinical and pathological characteristics.

Category	Characteristics	CR group (n=24)	NR group (n=26)	*P* value
Baseline	Age (years)	37 (± 14)	40 (± 16)	0.39
	Male (%)	9 (37.5)	15 (57.7)	0.15
	MAP (mmHg)	118.5 (± 14.7)	129.9 (± 16.5)	*0.013*
	BMI (kg/m^2^)	26.1 (± 3.8)	26.5 (± 5.2)	0.77
	Microscopic Hematuria (HPF)	5.8 (± 8.2)	132.3 (± 199.4)	*0.003*
	24hUTP (g/24h)	8.8 (± 3.8)	8.7 (± 4.6)	0.91
	Serum Albumin (g/L)	18.8 (± 4.0)	24.1 (± 4.2)	*<0.001*
	Serum Creatinine (μmol/L)	74.8 (± 26.8)	169.0 (± 151.2)	*0.004*
	eGFR (mL/min/1.73m^2^)	101 (± 29)	62 (± 35)	*<0.001*
	TCHO (mmol/L)	10.8 (± 4.0)	7.3 (± 2.5)	*<0.001*
	TG (mmol/L)	2.9 (± 1.4)	2.2 (± 1.4)	0.08
	Serum IgG (mg/dL)	573.9 (± 236.4)	714.1 (± 300.1)	0.08
	Serum IgA (mg/dL)	297.8 (± 130.6)	312.7 (± 170.0)	0.73
	Serum IgM (mg/dL)	181.2 (± 109.1)	104.0 (± 65.1)	*0.005*
	Serum C3 (mg/dL)	111.6 (± 28.0)	96.9 (± 25.6)	0.06
	Serum C4 (mg/dL)	27.7 (± 8.7)	25.2 (± 7.8)	0.30
Oxford classification	M 0/1	0/24	0/26	1.00
	E 0/1	21/3	5/21	*<0.001*
	S 0/1	23/1	15/11	*0.002*
	T 0/1/2	11/5/8	2/5/19	*0.005*
	C 0/1/2	22/2/0	10/6/10	*<0.001*
Immunofluorescence	IgA deposits (+)	2.3 (± 0.5)	2.6 (± 0.5)	*0.015*
	C3 deposits (+)	1.0 (± 1.0)	2.3 (± 0.8)	*<0.001*
	C1q deposits (%)	7 (29.2)	7 (26.9)	0.86
	IgG deposits (%)	11 (45.8)	10 (38.5)	0.60
	FRA deposits (%)	14 (62.5)	21 (80.8)	0.15

CR, complete remission; NR, non-remission; MAP, mean arterial pressure; BMI, body mass index; 24hUTP, 24-hour urinary total protein; eGFR, estimated glomerular filtration rate; TCHO, total cholesterol; TG, triglyceride.

**Figure 2:**
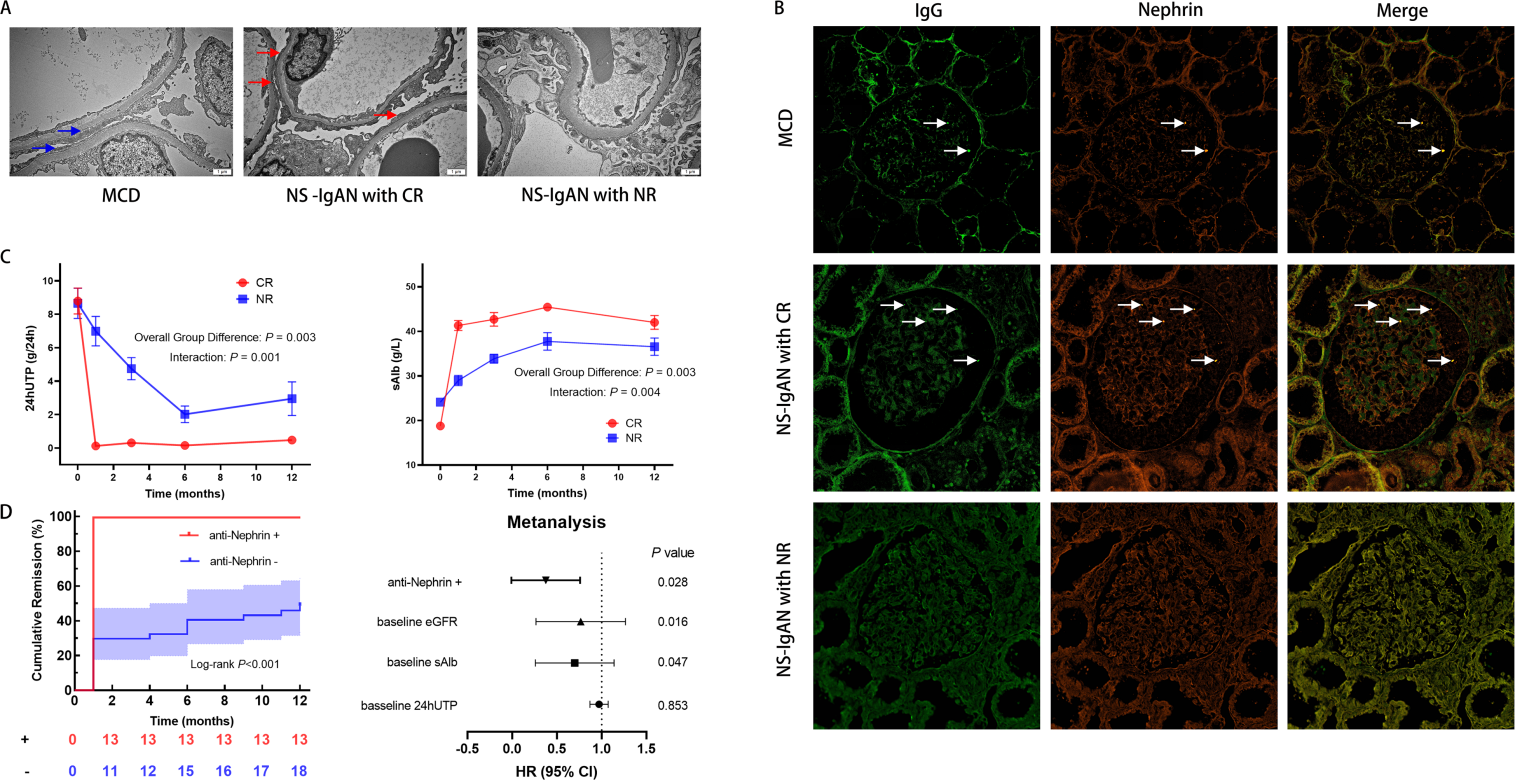
Clinicopathological features of IgA nephropathy with nephrotic syndrome (NS-IgAN). **(A)** Transmission electron microscopy (TEM) revealing extensive foot process effacement in the complete remission (CR) group (original magnification: ×15,000). **(B)** Confocal immunofluorescence analysis demonstrating IgG (green) colocalization with nephrin (red) (scale bar = 20μm). Specific IgG-nephrin colocalization (white arrows) was observed in minimal change disease (MCD) and CR groups, but absent in non-remission (NR) specimens. **(C)** Comparative longitudinal analysis of 24-hour urinary total protein (24hUTP) and serum albumin (sAlb) between CR and NR cohorts. **(D)** Kaplan-Meier curves for time to complete remission and multivariable Cox proportional hazards regression model (forest plot) identifying anti-nephrin positivity as an independent predictor of accelerated remission. eGFR, estimated glomerular filtration rate.

To determine morphology changes of podocytes of IgAN with NS, we used TEM. It’s generally acknowledged that MCD is characterized by an extensive fusion of podocyte foot processes, resulting in severe proteinuria. In patients of IgAN with NS, we also found the varying degrees of foot process fusion. In CR group, foot processes of neighboring podocytes were extensively interdigitated, similar to MCD, while for those still with heavy proteinuria after 3 months, the fusion was not so significant (**Figure 2A**). This might indicate a possibility of combination of MCD in some IgAN presenting NS.

### 3.3 Laser confocal immunofluorescence analysis

Laser scanning confocal immunofluorescence microscopy was used to further investigate the potential presence of an MCD-like phenotype. Three biopsy-confirmed MCD patients were selected as controls. In two of these cases, specific colocalization of nephrin with punctate IgG deposits was observed: Antigen specificity was confirmed by distinct spatial association between IgG and nephrin, which was absent with intracellular podocyte-specific proteins (synaptopodin, podocin, and WT1). Strikingly, an identical pattern of nephrin-IgG co-localization was identified in 54.2% (13/24) of patients in the CR group, mirroring the MCD phenotype. Conversely, this specific immunofluorescence signature was absent in all patients in the NR group (0%) (**Figure 2B**, **Supplementary Figure 1**).

### 3.4 Treatment response and renal outcomes

According to KDIGO guideline recommendations, all NS-IgAN patients received standardized treatment per MCD protocols unless contraindicated. No significant differences were observed in the utilization rates of corticosteroids (21/24 [87.5%] vs. 24/26 [92.3%]; *P* = 1.000), immunosuppressants (4/24 [16.7%] vs. 9/26 [34.6%]; *P* = 0.278), or RAASi (9/24 [37.5%] vs. 10/26 [38.5%]; *P* = 0.944) between the CR and NR groups, with detailed data for the PR group provided in the **Supplementary Materials**. Among patients receiving immunosuppressive therapy, cyclosporine was the most frequently prescribed agent (26.3% of treated cases).

We performed a *post-hoc* power analysis for our primary endpoint—the difference in CR rates between anti-nephrin positive and negative groups. Given the observed effect size (100% vs. 29.7% CR rate) and sample sizes (n1 = 13, n2 = 37), the statistical power exceeded 99.98% at α = 0.05. This indicates that our study was amply powered to detect this large and clinically significant difference.

During a median follow-up of 29 months (ranging from 12 months to 102 months), significant divergence in therapeutic trajectories was observed between the CR and NR cohorts. Repeated-measures ANOVA with false discovery rate (FDR) adjustment revealed statistically significant time × group interaction effects for both 24hUTP (F(4, 88) = 4.85, *P* = 0.001) and serum albumin (F(2.69, 40.41) = 270.09, *P* = 0.004), confirming fundamentally distinct patterns of change over time between the two groups (**Figure 2C**). These differences remained significant at multiple time points after FDR correction (adjusted *P* < 0.05), providing robust, false-discovery-controlled evidence that these parameters are strong discriminators of treatment response. In contrast, the eGFR analysis showed no statistically significant time × group interaction effect after FDR adjustment (F(4, 88) = 0.32, *P* = 0.865), indicating that the rate of renal function decline over the observation period was not significantly different between groups. However, a significant main effect of group was identified (F(1, 22) = 7.61, *P* = 0.011), indicating consistently higher eGFR levels in the CR group across all time points, despite parallel rates of decline (**Supplementary Figure 2**).

Kaplan-Meier analysis revealed a significant association between anti-nephrin antibody status and the time to achieve CR (**Figure 2D**). Patients positive for anti-nephrin antibody attained CR significantly faster than those in the antibody-negative group (Log-rank test, *P* < 0.001). Strikingly, all antibody-positive patients (13/13, 100%) achieved CR within 1 month of initiating treatment. In contrast, the majority of antibody-negative patients (26/37, 70.3%) failed to achieve CR at any point during 12 months. To adjust for potential confounding factors, a multivariable Cox proportional hazards regression model was employed, incorporating baseline 24hUTP, serum albumin, and eGFR. After these adjustments, anti-nephrin positivity persisted as a strong and independent predictor of accelerated CR (hazard ratio [HR] = 0.40, 95% confidence interval [CI]: 0.17–0.90, *P* = 0.028) (**Figure 2D**).

## 4 Discussion

NS represents a rare clinical manifestation IgAN, raising fundamental questions about its pathobiological classification: whether it constitutes a distinct IgAN subtype or coexists with MCD. Historical diagnostic challenges stemmed from the absence of reliable molecular markers for MCD differentiation. Recent identification of anti-nephrin autoantibodies as pathognomonic biomarkers for podocytopathies, including MCD, now enables mechanistic investigation of this clinical enigma.

As a core structural component of the podocyte slit diaphragm, nephrin maintains glomerular filtration barrier integrity through its extracellular domain forming a zipper-like molecular configuration (14). Studies have demonstrated that anti-nephrin antibodies induce podocyte injury by specifically binding to nephrin at the slit diaphragm, disrupting its spatial architecture and triggering foot process effacement with consequent heavy proteinuria (11). Hengel et al. (11) pioneered a quantitative assay for circulating anti-nephrin antibodies, revealing their significant correlation with proteinuria severity in MCD patients. Furthermore, Watts’ team (12) employed immunofluorescence co-localization techniques to demonstrate precise spatial overlap between punctate IgG deposits and nephrin expression in MCD renal biopsies, providing histopathological evidence for antibody-mediated podocytopathy. These pivotal discoveries not only elucidate MCD pathogenesis but also establish a conceptual framework for interpreting the steroid-sensitive NS-IgAN subtype characterized by anti-nephrin antibody co-localization and MCD-like therapeutic responsiveness observed in our cohort.

Our 15-year single-center retrospective analysis revealed a NS-IgAN prevalence of 9.0% (117/1,298), consistent with previously reported rates (6.7–14.7%) and challenging the notion of NS-IgAN as a rare entity (6–9). We identified a distinct clinical-pathological subset characterized by exhibiting features of both IgAN and MCD, with an estimated prevalence of this overlap syndrome of 35.8% (24/67) among NS-IgAN cases. This observation aligns with the findings of Roman et al. (15) regarding the association between nephrin autoantibodies and MCD-like morphology with steroid sensitivity. Anti-nephrin IgG colocalization was observed in 54.2% (13/24) of patients who achieved rapid CR, a rate comparable to regional MCD cohorts (13).

Notably, despite uniform treatment exposure across all patient groups in accordance with standardized KDIGO-recommended MCD protocols (unless contraindicated), therapeutic responses diverged markedly. This divergence suggests that outcomes are more likely attributable to underlying pathophysiological differences—particularly the presence of an IgAN-MCD overlap phenotype—rather than to heterogeneity in treatment regimens.

The sustained separation of eGFR curves, characterized by a persistent baseline deficit in the NR group—a feature more aligned with classic IgAN than MCD—reflects a pre-existing difference in initial renal function rather than a divergent rate of functional decline, as indicated by the non-significant time × group interaction effect. This suggests that while the 12-month follow-up period was sufficient to capture rapid biochemical responses, it may be inadequate to discern differences in long-term renal progression, which often unfolds over years or decades. Critically, our repeated-measures ANOVA with FDR adjustment confirmed statistically significant time × group interaction effects for both 24hUTP and serum albumin, underscoring fundamentally distinct temporal patterns of change between the CR and NR groups. Kaplan-Meier analysis further demonstrated a profound divergence in therapeutic response trajectories: all anti-nephrin antibody-positive patients (13/13) achieved rapid CR within 1 month, starkly contrasting with the treatment resistance observed in the majority of antibody-negative patients. This clear biological dichotomy was reinforced by multivariable Cox regression adjusting for key baseline confounders, which affirmed anti-nephrin positivity as a strong and independent predictor of accelerated time-to-remission. This strongly supports the potential clinical utility of this biomarker for identifying a steroid-hyperresponsive disease variant. While these results are compelling, they should be interpreted with caution due to the sample size constraints. Still, the HR point estimate and confidence interval (which remains entirely below 1.0) consistently support anti-nephrin antibody status as a strong predictive biomarker for accelerated treatment response, providing biologically plausible insights for future validation in larger cohorts. Nonetheless, the clinically meaningful response observed in a proportion (11/37, 29.7%) of antibody-negative patients implies the existence of additional steroid-responsive pathways beyond anti-nephrin status, indicating a more complex pathophysiology than a simple binary mechanistic dichotomy.

Although anti-nephrin antibodies show promise as molecular biomarkers for identifying IgAN-MCD overlap cases, this study has several key limitations. First, the unavailability of serum samples precluded quantification of circulating anti-nephrin autoantibody titers at renal biopsy, which would provide critical insights for clinical diagnostics. Second, the relatively short follow-up period may be insufficient to evaluate hard long-term renal outcomes in chronic nephropathies. Third, all enrolled patients were of Asian descent, and the absence of data from non-Asian populations limits the generalizability of our findings—external validation across diverse ethnic groups is required. In conclusion, research in this area remains exploratory. Further investigations with larger sample sizes and multiethnic cohorts are warranted to validate and extend these findings.

Urinary protein quantification is a well-established biomarker for prognostic evaluation and therapeutic guidance in IgAN, with its magnitude and persistence being strongly associated with the risk of progression to ESRD (16, 17). Identifying IgAN-MCD overlap pathology within NS-IgAN carries critical therapeutic implications: Patients with this dual diagnosis often achieve CR with brief courses of corticosteroids, thereby avoiding inappropriate prolonged immunosuppression (15). This distinction enables clinicians to optimize therapeutic strategies during renal biopsy interpretation, particularly by preventing unnecessary long-term high-intensity immunosuppression in steroid-hyperresponsive overlap cases. While renal biopsy remains the gold standard for diagnosing kidney diseases, it has inherent limitations and cannot resolve all diagnostic challenges, particularly in complex or overlapping conditions such as NS-IgAN. In light of our findings and consistent with emerging evidence, we propose implementing quantitative serum anti-nephrin autoantibody profiling combined with renal biopsy-based anti-nephrin-IgG immunofluorescence co-localization assays to establish an integrated diagnostic framework for NS-IgAN management. This combined approach enables precise identification of IgAN-MCD overlap cases through molecular subtyping, thereby guiding evidence-based personalized therapeutic regimens. By ensuring therapeutic efficacy while minimizing unnecessary prolonged exposure to high-dose corticosteroids or immunosuppressants, this strategy holds significant promise for improving long-term renal outcomes in patients.

## Data Availability

The original contributions presented in the study are included in the article/**Supplementary Material**. Further inquiries can be directed to the corresponding authors.
