# Supplementary material for: How to identify IgA nephropathy presenting as nephrotic syndrome coexisting with minimal change disease? A 15-year single-center clinicopathological analysis
Source: Front Immunol. 2025 Nov 6;16:1669276. doi: 10.3389/fimmu.2025.1669276 (PMC12631404; doi:10.3389/fimmu.2025.1669276)
Supplement: Supplementary file 1 [file Image1.pdf]

Supplementary Material

1 Supplementary Tables

Supplementary Table 1. Baseline clinical and pathological characteristics.

	Characteristics	CR group (n=24)	PR group (n=17)	NR group (n=26)
Baseline	Age (years)	37 (±14)	43 (±16)	40 (±16)
	Male (%)	9 (37.5)	9 (52.9)	15 (57.7)
	MAP (mmHg)	118.5 (±14.7)	125.1 (±15.9)	129.9 (±16.5)
	BMI (kg/m^2)	26.1 (±3.8)	25.5 (±3.4)	26.5 (±5.2)
	Microscopic Hematuria (HPF)	5.8 (±8.2)	4.1 (3.3, 132.5)	132.3 (±199.4)
	UTP (g/24h)	8.8 (±3.8)	7.3 (±2.2)	8.7 (±4.6)
	Serum Albumin (g/L)	18.8 (±4.0)	23.9 (±4.2)	24.1 (±4.2)
	Serum Creatinine (μmol/L)	74.8 (±26.8)	117.3 (±65.8)	169.0 (±151.2)
	eGFR (mL/min/1.73m^2)	101 (±29)	72 (±34)	62 (±35)
	TCHO (mmol/L)	10.8 (±4.0)	8.5 (±3.2)	7.3 (±2.5)
	TG (mmol/L)	2.9 (±1.4)	2.5 (±1.3)	2.2 (±1.4)
	Serum IgG (mg/dL)	573.9 (±236.4)	687.3 (±358.1)	714.1 (±300.1)
	Serum IgA (mg/dL)	297.8 (±130.6)	268 (229, 303.5)	312.7 (±170.0)
	Serum IgM (mg/dL)	181.2 (±109.1)	76.4 (66.5, 139)	104.0 (±65.1)
	Serum C3 (mg/dL)	111.6 (±28.0)	97.9 (±30.8)	96.9 (±25.6)
	Serum C4 (mg/dL)	27.7 (±8.7)	29.8 (±11.2)	25.2 (±7.8)

	Characteristics	CR group (n=24)	PR group (n=17)	NR group (n=26)
Oxford classification	M 0/1	0/24	0/17	0/26
	E 0/1	21/3	11/6	5/21
	S 0/1	23/1	9/8	15/11
	T 0/1/2	11/5/8	10/3/4	2/5/19
	C 0/1/2	22/2/0	11/5/1	10/6/10
Immunofluorescence	IgA deposits (+)	2.3 (±0.5)	2.6 (±0.7)	2.6 (±0.5)
	C3 deposits (+)	1.0 (±1.0)	1.9 (±1.0)	2.3 (±0.8)
	C1q deposits (%)	7 (29.2)	4 (23.5)	7 (26.9)
	IgG deposits (%)	11 (45.8)	5 (29.4)	10 (38.5)
	FRA deposits (%)	14 (62.5)	12 (70.6)	21 (80.8)

CR, complete remission; PR, partial remission; NR, non-remission; MAP, mean arterial pressure; BMI, body mass index; 24hUTP, 24-hour urinary total protein; eGFR, estimated glomerular filtration rate; TCHO, total cholesterol; TG, triglyceride.

2 Treatment of Partial Remission Group

All NS-IgAN patients in this cohort received standardized initial therapy per KDIGO guideline recommendations for MCD protocols unless contraindicated, with consistent treatment application across CR, PR, and NR groups. Medication utilization rates in the PR group (corticosteroids: 15/17, 88.2%; immunosuppressants: 4/17, 23.5%; RAASi: 7/17, 41.2%) showed no significant differences compared to either CR or NR groups (all $P > 0.05$).

This consistency in treatment exposure across all three groups strengthens our conclusion that the divergent therapeutic responses are more likely driven by underlying pathophysiological differences (i.e., the presence of an IgAN-MCD overlap phenotype) rather than by heterogeneity in treatment regimens.

3 Supplementary Figures

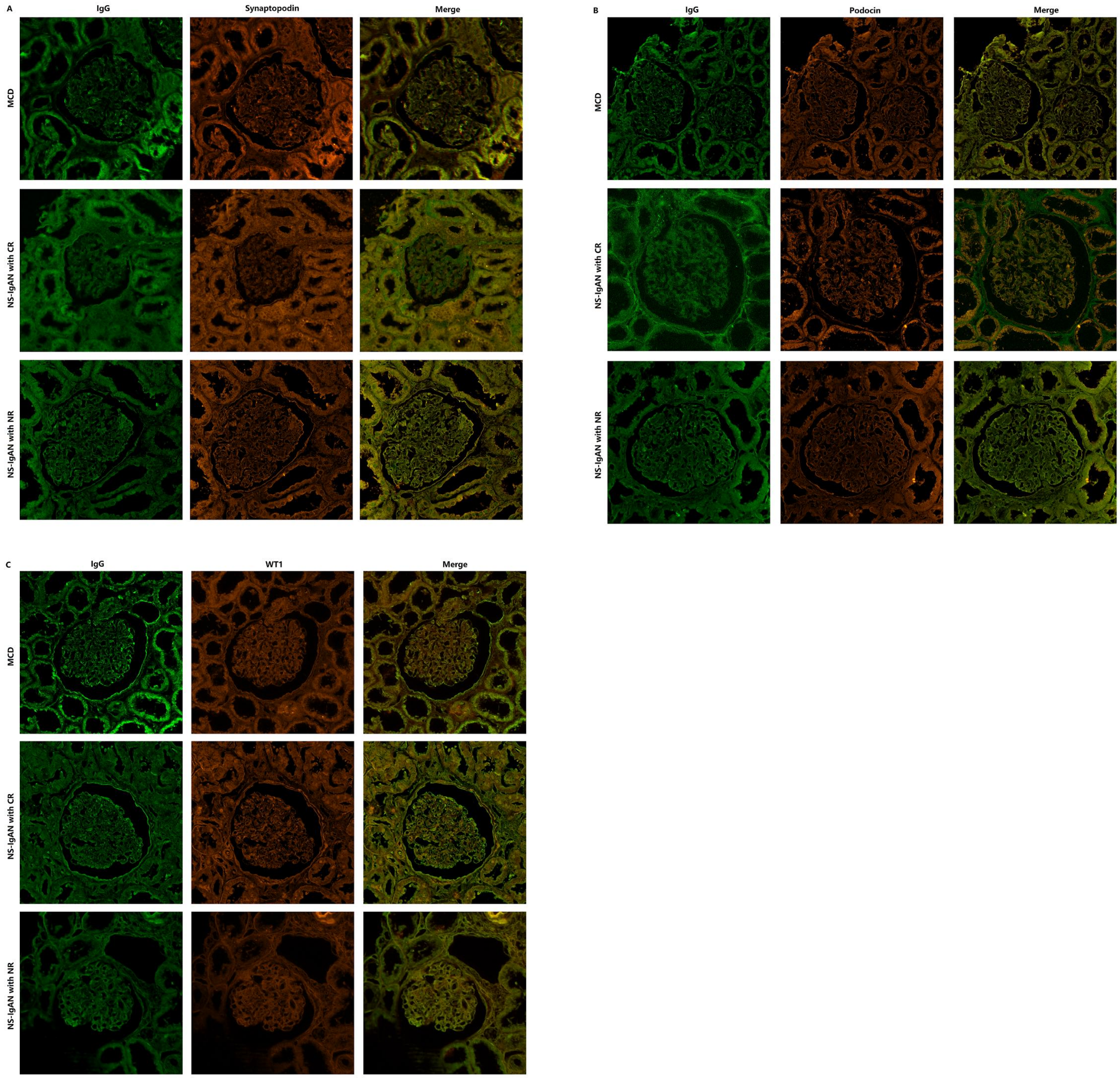

Supplementary Figure 1. Specificity of IgG colocalization with nephrin. Confocal immunofluorescence analysis demonstrating the absence of colocalization between IgG (green) and other podocyte-specific markers: A) synaptopodin (red), B) podocin (red), and C) WT1 (red). MCD, minimal change disease; NS-IgAN, IgA Nephropathy with Nephrotic Syndrome; CR, complete remission; NR, non-remission.

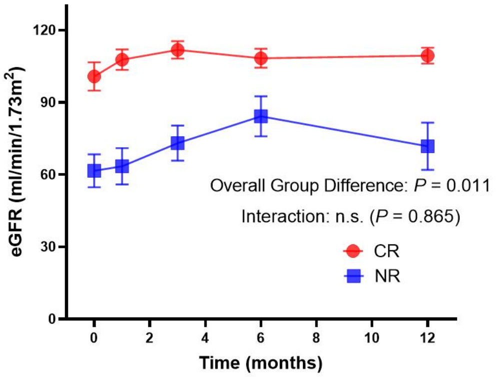

Supplementary Figure 2. Comparative longitudinal analysis of estimated glomerular filtration rate (eGFR) between complete remission (CR) and non-remission (NR) cohorts.
